# *In Utero* Exposure to Diethylstilbestrol and Blood DNA Methylation in Women Ages 40–59 Years from the Sister Study

**DOI:** 10.1371/journal.pone.0118757

**Published:** 2015-03-09

**Authors:** Sophia Harlid, Zongli Xu, Vijayalakshmi Panduri, Aimee A. D’Aloisio, Lisa A. DeRoo, Dale P. Sandler, Jack A. Taylor

**Affiliations:** 1 Epidemiology Branch, National Institute of Environmental Health Sciences, NIH, Research Triangle Park, North Carolina, United States of America; 2 Laboratory of Molecular Carcinogenesis, National Institute of Environmental Health Sciences, NIH, Research Triangle Park, North Carolina, United States of America; 3 Department of Global Public Health and Primary Health Care, University of Bergen, Bergen, Norway; Dartmouth Medical School, UNITED STATES

## Abstract

*In utero* exposure to diethylstilbestrol (DES) has been associated with increased risk of adverse health outcomes such as fertility problems and vaginal as well as breast cancer. Animal studies have linked prenatal DES exposure to lasting DNA methylation changes. We investigated genome-wide DNA methylation and *in utero* DES exposure in a sample of non-Hispanic white women aged 40–59 years from the Sister Study, a large United States cohort study of women with a family history of breast cancer. Using questionnaire information from women and their mothers, we selected 100 women whose mothers reported taking DES while pregnant and 100 control women whose mothers had not taken DES. DNA methylation in blood was measured at 485,577 CpG sites using the Illumina HumanMethylation450 BeadChip. Associations between CpG methylation and DES exposure status were analyzed using robust linear regression with adjustment for blood cell composition and multiple comparisons. Although four CpGs had p<10^5^, after accounting for multiple comparisons using the false discovery rate (FDR), none reached genome-wide significance. In conclusion, adult women exposed to DES *in utero* had no evidence of large persistent changes in blood DNA methylation.

## Introduction

The synthetic estrogen diethylstilbestrol (DES) was introduced as a drug to prevent miscarriage in the 1940’s and was widely prescribed to pregnant women. More than 2 million American women took the drug during pregnancy, exposing both themselves and their fetuses to high doses of estrogen [1]. Clinical trials during the 1950s failed to show any effect of DES on reducing the risk of miscarriage, and in 1971, DES exposure during fetal development was found to be associated with a rare form of vaginal adenocarcinoma in adolescent girls [2]. Since then, a number of other adverse health outcomes including infertility, early menopause and breast cancer, have been connected to *in utero* DES exposure [2–5].

Animal models show that early exposure to estrogen compounds, including DES, can change the expression levels of several genes [6–8]. Numerous animal studies have identified DES-exposure related epigenetic changes including DNA methylation and histone modifications [9–11]. While these estrogen-induced changes have mainly been observed in uterine or vaginal tissue, one study of the phytoestrogen genistein identified DNA-methylation changes in blood from 12-week old mice that had been exposed prenatally [7].

It is possible that prenatal exposure to DES induces lasting modifications to hematopoietic stem cells if the exposure occurs during early fetal development [12]. We hypothesized that exposure to DES *in utero* gives rise to persistent epigenetic changes (detectable in blood) and that these changes are partly responsible for the observed increased risk of infertility and cancer related health outcomes in the offspring. We conducted a study of DNA methylation patterns in whole blood from 100 women reporting that their mothers had used DES during pregnancy, and compared them to 100 women without exposure using the Illumina HumanMethylation450 BeadChip. To our knowledge this is the first study to evaluate possible effects of *in utero* DES exposure on genome-wide DNA methylation in humans.

## Materials and Methods

### Study design

All women in the study were participants of the Sister Study—a prospective cohort study of environmental and familial risk factors for breast cancer and other diseases. This cohort includes 50,884 women who were all breast cancer free at enrollment but had at least one sister diagnosed with breast cancer. All participants provided a blood sample at baseline and completed extensive questionnaires including a self-administered family history questionnaire on prenatal exposures. Detailed information about the Sister Study can be found at http://sisterstudy.niehs.nih.gov [13].

Within the Sister Study a sample of the participant’s mothers was also invited to participate in a sub study entitled the Mothers Validation Study (MVS). Sampling for the MVS was restricted to mothers under 60 who were reported to be alive at their daughters’ enrolment into the Sister Study and additionally weighted to include women whose daughters reported rare early life exposures, including DES. A total of 1802 mothers completed the MVS questionnaire which included a question about whether the she had used DES during pregnancy. The concordance between mothers and daughters reports on DES usage and exposure was addressed (S1 Table). In our sample selection we excluded 84 women with discordant mother-daughter reports on DES exposure, 163 with missing data on DES exposure, 39 women with insufficient blood samples, and 270 women because of age and ethnic restrictions (ages were limited to 40–59 years of age to capture the time period when DES was most widely prescribed and the sample was restricted to non-Hispanic whites because of possible racial/ethnic differences in methylation). Of the remaining women (n = 1246), with concordant mother-daughter DES reports, we randomly selected 100 of the 125 exposed women and 100 of the 1121 unexposed women (frequency matched by age to the exposed women) (S1 Fig.). This sample set has previously been used to study the effects of smoking on DNA methylation in blood [14].

### Ethics Statement

Informed written consent was obtained from all participants prior to participation. The study was approved by the Institutional Review Boards of the National Institute of Environmental Health Sciences (NIEHS), National Institutes of Health, and the Copernicus Group (http://www.cgirb.com/irb-services/).

### DNA extraction, bisulfite conversion and quality control

All blood samples were collected using glass blood collection tubes with acid citrate dextrose (ACD). This was done during a home visit at baseline and the blood was stored at-20C prior to DNA extraction. Genomic DNA was extracted using automated equipment (Autopure LS, Qiagen, Valencia, CA) and quantified with Quant-iT PicoGreen dsDNA reagent (Invitrogen). One microgram (μg) of extracted DNA was bisulfite converted using the EZ DNA methylation kit (Zymo Research, Irvine, CA) according to the manufacturer’s protocol.

### Illumina 450K methylation array

Methylation analysis was conducted at the NIEHS Molecular Genomics Core using the Illumina HumanMethylation450 BeadChip (Illumina, San Diego, CA). Samples were randomly assigned to eighteen beadchips with 12 spots on each using a balanced design with respect to exposure and age (<50 vs >+50). We included 6 DNA methylation controls (Zymo Research, Irvine, CA) and 10 duplicate samples, which were placed on different chips. One of the duplicates was eventually excluded due to poor methylation data quality. For the remaining 9 pairs of duplicates we calculated Pearson’s correlation coefficients for Infinium I and Infinium II probes separately (S2 Table). DNA was hybridized to the array following the manufacturer’s protocol and then scanned with an Illumina iScan (Illumina, San Diego CA). Raw intensity data were extracted using Illumina GenomeStudio software (version 2011.1).

We excluded 48,494 CpG probes based on the following criteria: 1) CpG probes with a common SNP (minor allele frequency ≥ 0.05 in Europeans based on the 1000 Genomes project data-release/20130502) located at the target CpG site; 2) CpGs with probes mapping to multiple genomic locations; 3) CpGs located on the Y chromosome.

At each CpG site on the array, methylation status was determined based on intensity measures of probes corresponding to unmethylated (*U*) or methylated (*M*) CpGs. We pre-processed the *M* and *U* intensity values separately for Infinium I and II CpG probes detailed as follows: 1) for Infinium I probes, we separately background corrected (using the Robust Multichip Average (RMA) method [15]) and quantile normalized red and green channel probes; 2) for Infinium II probes, we first corrected dye bias between *U* and *M* intensity values using the normalizeMethyLumiSet method in the R package “methylumi”, and then performed RMA background correction and quantile normalization separately for *U* and *M* intensity values; 3) The methylation level (beta value) for each CpG site was calculated as the ratio of normalized fluorescent intensities between methylated and unmethylated alleles *β = M/(M+U+100)*.

The DNA methylation data set used in this publication can be accessed via www.sisterstudystars.org, or by contacting one of the authors.

### Previously reported candidate genes affected by DES

We also specifically report on nine genes (*EMB*, *WNT11 TGFB1*, *ERBB2*, *EGFR*, *LTF*, *EGF*, *FOS*, and *JUN*) that have previously been shown to have differential gene expression in mice uterine or vaginal tissue following pre or perinatal exposure to DES [8, 11, 16–20]. We compared the mean β-value for 75 CpGs in the 5’ region (as annotated by Illumina) of these genes for women with and without DES exposure histories.

### Statistical analysis

Robust linear regression was used to test the association between CpG methylation profiles (β-values) and DES exposure status. In order to adjust for multiple testing, the false discovery rate (FDR) was calculated using the q-value framework, q<0.05 was used as the cutoff for genome wide significance [21]. In the association tests we adjusted for the proportions of 6 different types of white blood cells (CD8 T cells, CD4 T cells, B cells, granulocytes, monocytes, and Natural killer cells) estimated using a method described by Houseman et al [22]. Briefly, we first utilized a publically available Illumina HumanMethylation450 dataset (GSE35069) [23] for 60 cell type specific samples to select the top 100 most informative CpGs with respect to blood cell types; based on methylation profiles of the 100 CpGs for our samples, we then estimated the composition of the 6 cell types in each sample. Additional adjustments were made for age at menarche, BMI, and parity.

## Results

### Participant characteristics

DES-exposed women were similar to unexposed women in regard to age, ever use of OCs, and smoking status; a greater percent of exposed women had BMI<25, menarche at 14+ years, and were nulliparous than unexposed women (Table 1).

**Table 1:** Baseline characteristics of DES-exposed and unexposed women selected from the Sister Study.

Characteristics	Exposed (n = 100)%	Unexposed (n = 100%)	P^d^
40–49 yr	42	42	
50–59 yr	58	58	1.00
Body Mass Index			
<20	7	7	
20–24.9	49	38	
25–29.9	26	25	
≥30	18	30	0.22
Age at Menarche			
<12 yr	15	25	
12–13 yr	51	49	
≥14 yr	34	26	0.17
Age at Menopause^a^			
<45 yr	11	8	
45–55yr	29	31	
≥55 yr	2	1	0.66
Menopause Status			
Postmenopausal	45	45	
Premenopausal	55	55	1.00
Oral Contraceptive Use			
Ever	90	92	
Never	10	8	0.62
HRT Use^b^			
Current	8	13	
Ever	15	12	
Never	22	19	0.42
Parity			
0	40	21	
1	13	16	
2	29	38	
≥3	18	25	0.03
Smoking Status^c^			
Current	8	4	
Former	35	34	
Never	54	58	0.60
Mothers Education at Age 13^c^			
<HS, HS/GED	55	60	
Some College	12	10	
Associate/Tech	12	9	
Bach degree	16	17	
Grad degree	4	2	0.82
Family Income During Childhood			
Well Off	9	9	
Middle Income	75	69	
Low Income	13	17	
Poor	3	5	0.73
Maternal Smoking^c^			
Definitely	34	32	
Probably	5	6	
Probably not	4	2	
Definitely not	56	59	0.83
Birth Weight^c^			
<2500 g	13	6	
2500-<4000g	70	71	
4000+ g	7	9	0.25
Gestational Age^c^			
Not Early, 2+Weeks	67	68	
Early, 2–4 Weeks	10	5	
Early, 1+ months	6	4	0.40

^a^Age at menopause was ascertained among the 45 exposed and 45 unexposed women who were postmenopausal

^b^HRT was ascertained among postmenopausal women only

^c^Column % does not add to 100% due to missing data

^d^Based on Chi-Square test

### Epigenome wide study

**Fig 1:**
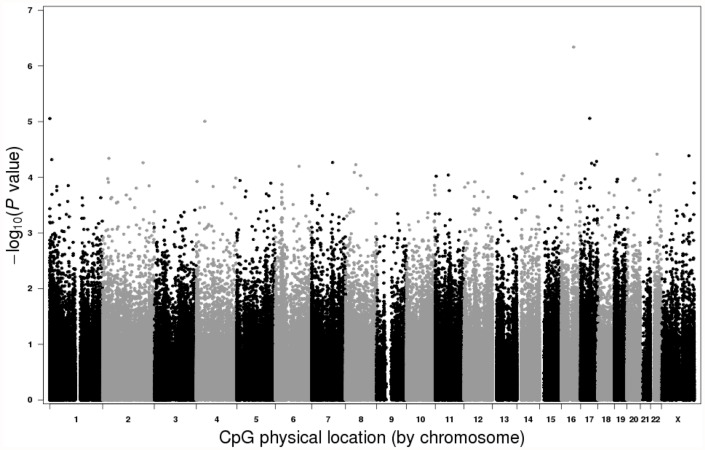
Manhattan plot. Log_10_ transformed DES association p-values for individual CpGs are plotted in relation to their chromosome location. No CpGs reached genome wide significance.

In our primary model, adjusted for multiple testing and blood cell composition, four CpGs in two different genes (*KIFC3*, and *DCAKD*) had nominal p values < 10^-5^, and an additional 18 CpGs in 18 different genes had nominal p values < 10^-4^ (Fig. 1). None of these 22 CpGs achieved genome-wide significance after considering multiple comparisons (q<0.05). Additional adjustments for age at menarche, BMI and parity did not affect the results.

### Candidate gene comparisons

**Fig 2:**
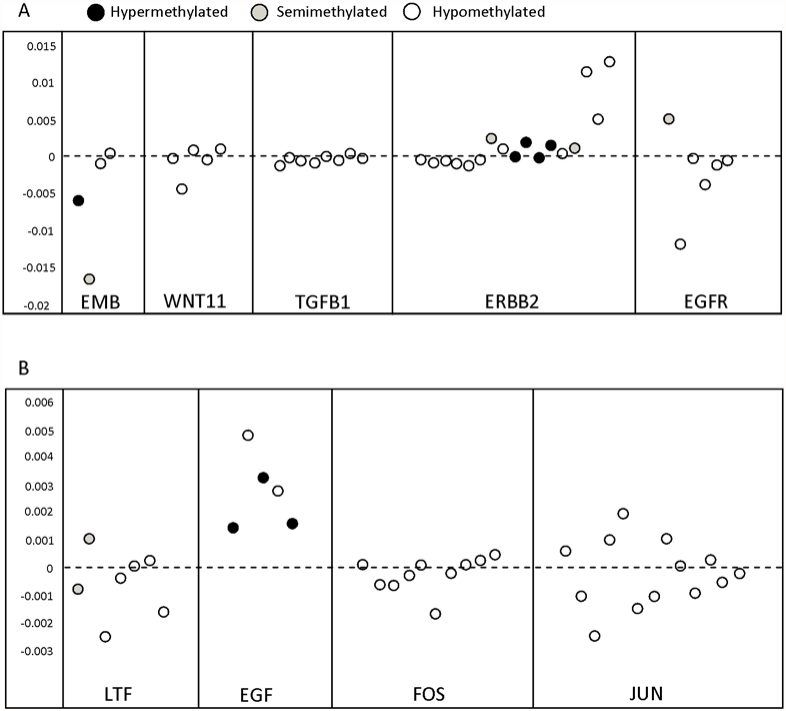
Distribution of CpGs in the 5’end of 9 genes associated with differential methylation in animal models exposed to DES. All CpGs are ordered from 5’ to 3’. Y axis shows the difference in mean beta values of exposed and unexposed women. Circle color depicts methylation status of individual CpGs (Black = Hyper-methylated [≥70%], Grey = Semi-methylated [<70%>30%], White = Hypo-methylated [≤30%]). All sites were selected based on annotations from the Illumina manifest. A) Genes that are up regulated in mice exposed to DES. B) Genes that are down regulated in mice exposed to DES.

We examined DNA methylation values at 75 CpG sites located in the 5’ regions of nine previously reported genes. At these sites the average differences in DNA methylation between exposed and unexposed women were < 1%. None of the 75 CpG sites remained significant after adjusting for multiple comparisons (Fig. 2 and S3 Table).

## Discussion

Epidemiologic studies have shown that women exposed to the synthetic estrogen DES *in utero* have increased risk of breast and vaginal cancer, preterm delivery, infertility, early menarche and other health outcomes[2, 4, 5, 24], and some of these risks, specifically concerning breast and vaginal cancer, persist as women grow older [3]. Although we hypothesized that persistent DNA methylation changes might be detectable in blood from women exposed to DES *in utero*, we found no evidence of this in our sample of non-Hispanic white women aged 40–59 years.

Several studies have found differences in gene expression and/or DNA methylation in adult mice or rats exposed to DES during development or early life [8, 17, 25]. Hyper or hypo-methylation of the 5’ promoter regions has previously been shown to control gene expression [26] which led us to focus on CpGs in this region of the gene. However, for 75 CpGs in the 5’ regions of the nine genes identified in rodent studies, we found no significant differences between exposed and unexposed women.

Strengths of this study include the availability of both mothers and daughters reports on DES exposure, and genome-wide coverage of CpG methylation sites (representing ~ 2% of CpGs in the genome). Limitations include lack of detailed information on dose, route of exposure, timing, or duration of DES use. Additionally we had limited power to detect small effect sizes (defined by the absolute methylation difference between two groups, divided by the standard deviation). With a sample size of 200 (1:1 case/control ratio) and assuming a p-value threshold of 10^-8^, our study had 80% power to detect CpG sites with effect sizes of at least 0.67. The average effect size of our top 4 CpG sites was 0.5, which would require a study sample size of 354 to obtain 80% power.

In this study we used DNA from blood rather than from a known target tissue such as vagina, cervix or endometrium. Histologic changes in target tissues, including vaginal epithelium, are known to correlate with dose of DES exposure and with increased risk of adverse outcomes such as vaginal carcinoma [3, 27]. Although it may be possible to compare methylation in target tissues using archival formalin-fixed paraffin-embedded (FFPE) samples, it is difficult to find adequate FFPE samples from DES-exposed women. In addition, methylation analysis of degraded and cross-linked FFPE samples present substantial technical challenges. In conclusion, although we cannot rule out the possibility of effects at younger ages or in other tissues, our study finds no evidence of large persistent effects of *in utero* DES exposure on blood DNA methylation.

## Supporting Information

S1 Fig Flowchart of the sample selection. The 100 exposed women were randomly selected from the 125 eligible exposed women. The 100 unexposed women were selected based on age frequency matching to the exposed women.
(PDF)

S1 Table Comparison of mother and daughter reports of in utero DES exposure between participants in the Mother’s Validation Study and The Sister Study. (PDF)

S2 Table Pearson’s correlation coefficients between duplicates using methylation beta values for Infinium I and Infinium II probes. (PDF)

S3 Table CpGs from 9 genes previously implicated to respond to DES exposure in animal models. (PDF)
