# Supplementary figures and images for: In Utero Exposure to Diethylstilbestrol and Blood DNA Methylation in Women Ages 40–59 Years from the Sister Study
Source: PLoS One. 2015 Mar 9;10(3):e0118757. doi: 10.1371/journal.pone.0118757 (PMC4353728; doi:10.1371/journal.pone.0118757)

**Figure S1:**

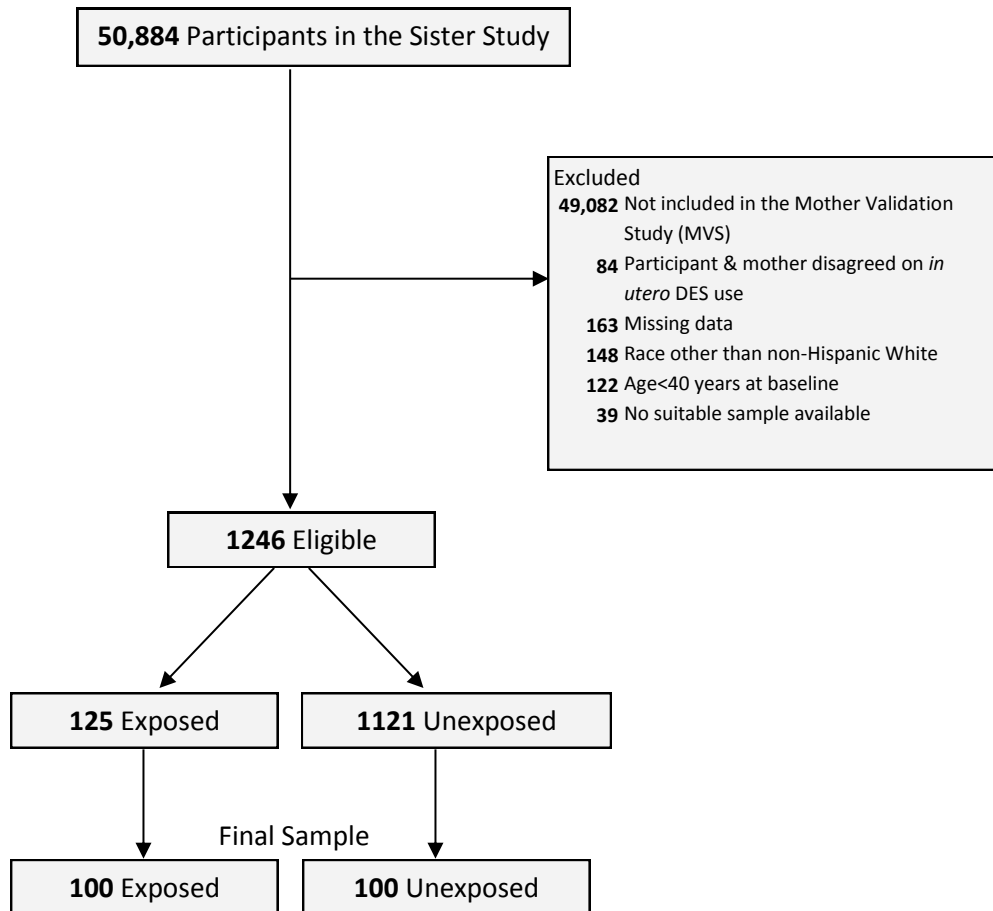

Supplement: S1 Fig — The 100 exposed women were randomly selected from the 125 eligible exposed women. The 100 unexposed women were selected based on age frequency matching to the exposed women. (PDF) [file pone.0118757.s001.pdf]
