# Supplementary material for: In Utero Exposure to Diethylstilbestrol and Blood DNA Methylation in Women Ages 40–59 Years from the Sister Study
Source: PLoS One. 2015 Mar 9;10(3):e0118757. doi: 10.1371/journal.pone.0118757 (PMC4353728; doi:10.1371/journal.pone.0118757)
Supplement: S1 Table — (PDF) [file pone.0118757.s002.pdf]

**Table S1.** Comparison of mother and daughter reports of *in utero* DES exposure between participants in the Mother's Validation Study and The Sister Study.

| **Mother's report:** When pregnant with your daughter did you take DES (diethylstilbestrol)?). | **Daughter's report:** During her pregnancy with you, did your mother take DES (diethylstilbestrol)?). | | TOTAL |
|---|---|---|---|
| | **Definitely/Probably YES** | **Definitely/Probably NO** | |
| **YES** | 139 | **6** | 145 |
| **NO** | **78** | 1416 | 1494 |
| TOTAL | 217 | 1422 | 1639[a] |

[a] Missing data: 163
