# Supplementary material for: In Utero Exposure to Diethylstilbestrol and Blood DNA Methylation in Women Ages 40–59 Years from the Sister Study
Source: PLoS One. 2015 Mar 9;10(3):e0118757. doi: 10.1371/journal.pone.0118757 (PMC4353728; doi:10.1371/journal.pone.0118757)
Supplement: S2 Table — (PDF) [file pone.0118757.s003.pdf]

Table S2. Pearson's correlation coefficients between duplicates using methylation beta values for Infinium I and Infinium II probes

Duplicates id	**Infinium I** correlation coefficients	**Infinium II** correlation coefficients
1	0.998689	0.998351
2	0.998613	0.997843
3	0.998898	0.998502
4	0.998827	0.99826
5	0.998564	0.997701
6	0.998794	0.998453
7	0.998896	0.998215
8	0.998399	0.99781
9	0.998351	0.997148
