# Supplementary material for: In Utero Exposure to Diethylstilbestrol and Blood DNA Methylation in Women Ages 40–59 Years from the Sister Study
Source: PLoS One. 2015 Mar 9;10(3):e0118757. doi: 10.1371/journal.pone.0118757 (PMC4353728; doi:10.1371/journal.pone.0118757)
Supplement: S3 Table — (PDF) [file pone.0118757.s004.pdf]

Table S3. CpGs from 9 genes previously implicated to respond to DES exposure in animal models

Gene	Probe	Chr	Position (Build 37)	Mean β-value (Unexposed)	Mean β-value (Exposed)	p-value	q-value
EMB	cg06928983	5	49738016	0.859357	0.853659	0.0947	0.8041
	cg19459207	5	49737773	0.545413	0.528859	0.2072	0.8337
	cg14497673	5	49737349	0.032618	0.031997	0.0422	0.7799
	cg02344943	5	49737236	0.015736	0.016116	0.4556	0.8778
WNT11	cg25904812	11	75918630	0.022216	0.022267	0.9955	0.9296
	cg22312494	11	75918089	0.065252	0.060821	0.0762	0.7968
	cg14860397	11	75917982	0.027419	0.028385	0.2368	0.8414
	cg17583449	11	75917844	0.172250	0.172342	0.8749	0.9213
	cg23400002	11	75917724	0.113947	0.114874	0.6882	0.9049
TGFB	cg24767336	19	41860095	0.017060	0.015846	0.3123	0.8567
	cg20410381	19	41860082	0.037171	0.037167	0.7848	0.9138
	cg23275502	19	41860019	0.006775	0.006504	0.3533	0.8627
	cg04547554	19	41860013	0.018561	0.017878	0.3331	0.8600
	cg05637188	19	41860004	0.008961	0.009146	0.5430	0.8890
	cg11714801	19	41859555	0.054915	0.054771	0.8401	0.9186
	cg13464744	19	41859304	0.011919	0.012203	0.3212	0.8583
	cg01107031	19	41859159	0.031890	0.031858	0.9203	0.9244
ERBB2	cg22632017	17	37844365	0.010688	0.010274	0.7436	0.9104
	cg12222323	17	37844398	0.016529	0.016259	0.1687	0.8248
	cg27052442	17	37844412	0.035288	0.034859	0.3411	0.8614
	cg25582403	17	37844421	0.079962	0.079341	0.7414	0.9103
	cg02330892	17	37844476	0.026459	0.025830	0.1385	0.8162
	cg19752722	17	37844579	0.016663	0.016385	0.2576	0.8441
	cg20007836	17	37844870	0.446764	0.449451	0.9307	0.9252
	cg26041593	17	37844971	0.110003	0.110941	0.7383	0.9099
	cg02023717	17	37846697	0.922984	0.922464	0.8444	0.9188
	cg04936632	17	37851022	0.876942	0.878250	0.6730	0.9033
	cg08585669	17	37853032	0.962175	0.961947	0.7008	0.9060
	cg10713339	17	37854801	0.867740	0.869198	0.1882	0.8302
	cg12413918	17	37855819	0.188375	0.188710	0.9414	0.9260
	cg22778981	17	37856133	0.376290	0.387347	0.0298	0.7699
	cg23731030	17	37856217	0.205060	0.211213	0.0794	0.7988
	cg23333072	17	37856228	0.271819	0.285360	0.0040	0.7298
	cg02433278	17	37856230	0.299715	0.304477	0.2157	0.8358
EGFR	cg16751451	7	55086091	0.535127	0.540411	0.3161	0.8575
	cg22396409	7	55086146	0.176531	0.165045	0.1205	0.8128
	cg07311521	7	55086164	0.037320	0.037207	0.7356	0.9096
	cg03860890	7	55086288	0.158881	0.155569	0.5327	0.8881

	cg05064645	7	55086868	0.014968	0.013893	0.2412	0.8416
	cg14094960	7	55086890	0.054493	0.053780	0.9820	0.9285
LTF	cg10749572	3	46506864	0.609295	0.608550	0.8136	0.9163
	cg00716083	3	46506554	0.307981	0.309074	0.7963	0.9149
	cg26307117	3	46506552	0.246023	0.243544	0.6017	0.8955
	cg27314002	3	46506519	0.153612	0.153324	0.8180	0.9167
	cg03933131	3	46506483	0.037499	0.037568	0.5867	0.8941
	cg12295918	3	46506481	0.042403	0.042708	0.9324	0.9253
	cg17527798	3	46506404	0.143154	0.141585	0.5780	0.8929
EGF	cg18905856	4	110832806	0.962001	0.963647	0.0419	0.7799
	cg11407540	4	110833060	0.990016	0.991486	0.3976	0.8690
	cg13379236	4	110833808	0.776560	0.779834	0.8387	0.9185
	cg12093976	4	110833884	0.128271	0.133086	0.0356	0.7745
	cg18195130	4	110833944	0.090867	0.093782	0.1368	0.8161
FOS	cg03509965	14	75743987	0.009940	0.010055	0.9691	0.9279
	cg11872076	14	75744010	0.007996	0.007484	0.1873	0.8298
	cg18717355	14	75744458	0.033672	0.033145	0.1987	0.8324
	cg07159858	14	75744601	0.026596	0.026348	0.5564	0.8907
	cg13819869	14	75745020	0.020663	0.020816	0.5757	0.8926
	cg14102251	14	75745098	0.039221	0.037600	0.3161	0.8575
	cg15337055	14	75745358	0.007369	0.007144	0.3905	0.8678
	cg25975379	14	75745397	0.008905	0.009024	0.5979	0.8951
	cg00773696	14	75745453	0.008477	0.008822	0.1067	0.8091
	cg20901874	14	75745470	0.021344	0.021791	0.2180	0.8361
JUN	cg25613251	1	59249811	0.004122	0.004311	0.4668	0.8799
	cg24059741	1	59249828	0.088431	0.089387	0.3798	0.8662
	cg07018071	1	59249834	0.024765	0.023770	0.1307	0.8148
	cg15096815	1	59249838	0.014172	0.014485	0.6141	0.8968
	cg03256465	1	59249883	0.019202	0.020205	0.2312	0.8396
	cg01909487	1	59249911	0.027892	0.026471	0.0846	0.8006
	cg26456528	1	59250203	0.022095	0.021922	0.8132	0.9163
	cg15500320	1	59250642	0.005928	0.006512	0.0931	0.8030
	cg15995771	1	59250858	0.010241	0.009209	0.5293	0.8877
	cg24425829	1	59250882	0.025686	0.027669	0.1677	0.8246
	cg15684811	1	59250924	0.033639	0.031154	0.2307	0.8396
	cg08444830	1	59250939	0.047362	0.046521	0.3181	0.8577
	cg20787340	1	59250966	0.011718	0.011184	0.1798	0.8274
